# Prevalence and molecular characterization of *Enterocytozoon bieneusi* and a new *Enterocytozoon* sp. in pet hairless guinea pigs (*Cavia porcellus*) from China[Fn FN1]

**DOI:** 10.1051/parasite/2023041

**Published:** 2023-09-20

**Authors:** Chaochao Lv, Chen Li, Jingsong Wang, Weifeng Qian

**Affiliations:** College of Animal Science and Technology, Henan University of Science and Technology No. 263 Kaiyuan Road, Luolong District Luoyang 471003 PR China

**Keywords:** *Enterocytozoon bieneusi*, new *Enterocytozoon* sp., Hairless guinea pigs, ITS, SSU rRNA gene, China

## Abstract

*Enterocytozoon bieneusi*, the most common microsporidian species, has been detected in humans and a variety of animals worldwide. However, limited information is available on the prevalence and molecular characterization of this parasite in guinea pigs. In this study, we conducted the first investigation of *E. bieneusi* infection in hairless guinea pigs recently introduced into China as new exotic pets. A total of 324 fecal samples were collected from hairless guinea pigs from a pet market and four breeding facilities in China. Sequence alignment of the internal transcribed spacer (ITS) revealed an infection rate of 14.2% (46/324) and two known ITS genotypes, S7 and PGP. Genotype S7 was the dominant genotype in these animals (42/46, 91.3%). Due to significant ITS sequence divergence, four and two PGP isolates from hairless and regular guinea pigs, respectively were further identified by PCR and phylogenetic analysis based on the small subunit (SSU) rRNA gene, as well as phylogenetic analysis of the ITS locus using *E. hepatopenaei* and two related genera *Enterospora* and *Nucleospora* as the outgroup. Three out of the six PGP isolates were successfully sequenced and generated the same sequences. Phylogenetic analysis of SSU rRNA and ITS loci revealed that PGP isolates formed a separate clade that was distinct and far away from *E. bieneusi*, suggesting that they represent a new species of *Enterocytozoon*. These findings indicate the dominance of zoonotic *E. bieneusi* genotype S7 in hairless guinea pigs and the existence of a cryptic *Enterocytozoon* species in guinea pigs.

## Abstract (fr)

*Enterocytozoon bieneusi*, l’espèce de microsporidies la plus commune, a été détectée chez les humains et chez divers animaux à travers le monde. Cependant, peu d’informations sont disponibles sur la prévalence et la caractérisation moléculaire de ce parasite chez le cobaye. Dans cette étude, nous avons mené la première enquête sur l’infection par *E. bieneusi* chez des cobayes sans poils récemment introduits en Chine en tant que nouveaux animaux de compagnie exotiques. Trois cent vingt-quatre échantillons fécaux ont été collectés sur des cobayes sans poils provenant d’un marché d’animaux de compagnie et de quatre établissements d’élevage en Chine. L’alignement des séquences de l’espaceur transcrit interne (ITS) a révélé le taux d’infection (14,2 %, 46 / 324) et deux génotypes ITS connus (S7 et PGP). Le génotype S7 était le génotype dominant chez ces animaux (42 / 46, 91,3 %). En raison d’une divergence significative des séquences ITS, quatre et deux isolats de PGP provenant respectivement de cobayes sans poils et ordinaires ont été identifiés par PCR, analyse phylogénétique basée sur le gène de l’ARNr de la petite sous-unité (SSU), ainsi que par analyse phylogénétique du locus ITS en utilisant *E. hepatopenaei* et deux genres apparentés *Enterospora* et *Nucleospora* comme groupe externe. Trois des six isolats de PGP ont été séquencés avec succès et ont généré les mêmes séquences. L’analyse phylogénétique de l’ARNr SSU et des loci ITS a révélé que les isolats de PGP formaient un clade distinct et éloigné de *E. bieneusi*, ce qui suggère qu’ils représentent une nouvelle espèce d’*Enterocytozoon*. Ces résultats indiquent la domination du génotype zoonotique *E. bieneusi* S7 chez les cobayes sans poils et l’existence d’une espèce cryptique d’*Enterocytozoon* chez les cobayes.

## Introduction

Microsporidia are obligate intracellular eukaryotic fungi that infect a wide range of vertebrate and invertebrate hosts. Currently, there are at least 1,700 species within more than 220 genera of microsporidia [8]. Among them, 17 species can infect humans. *Enterocytozoon bieneusi* is the most common microsporidian species in humans, accounting for more than 90% of cases of human microsporidiosis [28]. Humans and animals acquire infection via fecal-oral transmission of spores from infected hosts through direct contact or by ingestion of contaminated water or food [14]. *Enterocytozoon bieneusi* infection can lead to self-limiting diarrhea, malabsorption, and wasting in immunocompetent individuals; life-threatening diarrhea can occur in immunocompromized individuals, such as AIDS patients and organ transplant recipients [15].

Genotyping tools based on the ribosomal internal transcribed spacer (ITS) have been widely used, and more than 500 genotypes of *E. bieneusi* have been identified from various hosts [14]. These genotypes were classified into 11 major groups by phylogenetic analysis [14]. Groups 1 and 2 comprise most potential zoonotic genotypes; whereas the other groups (Groups 3–11) appear to be more host-adapted [14]. To date, more than 100 *E. bieneusi* genotypes have been identified from wild, laboratory and pet rodents worldwide [7, 9, 11, 13, 15, 16, 18, 21, 24–27, 31–33, 35–37]. There are limited molecular data on this parasite from guinea pigs (*Cavia porcellus*). To date, only three studies have focused on the molecular characterization of *E. bieneusi* in household, pet, and laboratory guinea pigs in Peru and China, and three ITS genotypes (Peru16, S7, and PGP) have been identified [2, 26, 27]. S7 is the most frequently found genotype in guinea pigs.

**Figure 1:**
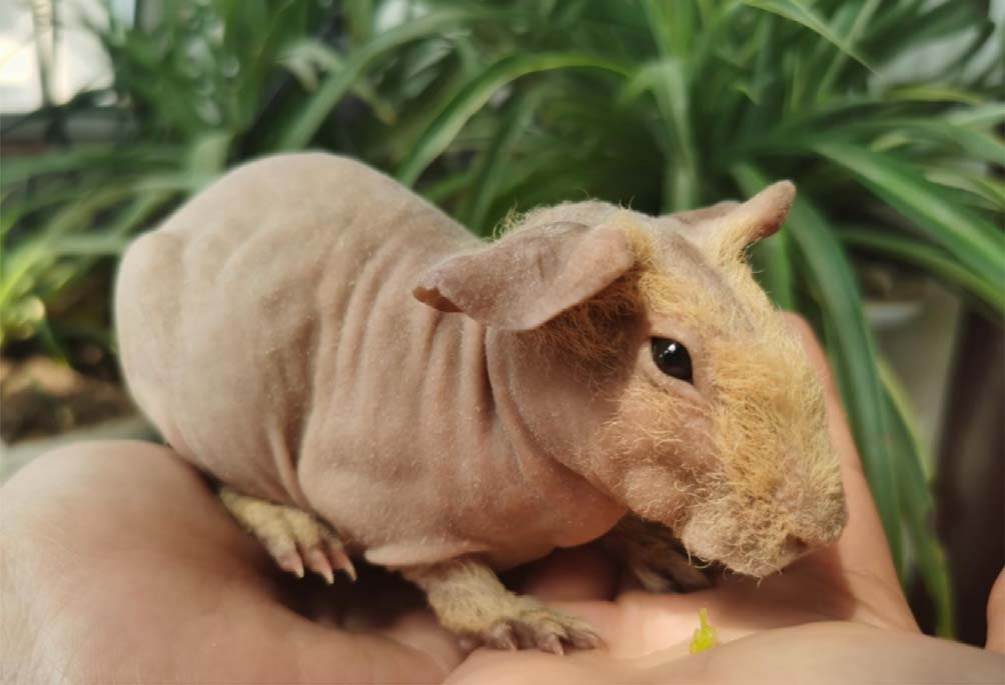
Hairless guinea pigs, called “Skinny pigs”, typically have hair on the muzzles, feet and legs, but are hairless over the remainder of the body.

The hairless guinea pig, also known as the “Skinny pig”, is an almost hairless breed originating from the laboratory (Fig. 1). It has a short history and is usually used for dermatological studies [23, 29]. It was introduced into the pet trade in the 1990s and was recently introduced into China as a pet animal. Pet rodents can serve as a source of many zoonotic pathogens, including viruses, bacteria, and parasites [17]; zoonotic transmission of *Enterocytozoon bieneusi* from domestic guinea pigs to a child in Peru has been reported [2]. There have been only two literature reports on the molecular characterization of *E. bieneusi* in regular guinea pigs from China [26, 27]. However, no data are available on this parasite in the new exotic animals in China. The purpose of this study was to determine the prevalence and zoonotic potential of *E. bieneusi* in pet hairless guinea pigs in China.

## Materials and methods

### Ethics statement

The research protocol was reviewed and approved by the Research Ethics Committee of Henan University of Science and Technology (approval No. 20180603). Collection of fecal samples was carried out with the consent of the animal’s owners.

### Sample collection

Between September 2018 and November 2019, fecal samples were collected from a total of 324 hairless guinea pigs from a pet market and four breeding facilities in four provinces of China (see Table 1 for details). The hairless guinea pigs in the pet market and breeding facilities were all pets offered for sale. These animals in the breeding facilities were raised in wire or plastic cages (4–12 animals per cage) and fed with pelleted diets supplemented by hay and green vegetables. In breeding facilities 1, 2 and 4, hygiene conditions were good; cages were cleaned once or twice weekly. In breeding facility 3, hygiene conditions were suboptimal, with infrequent cleaning (every 2 weeks) and poor ventilation. All the animals examined were kept in separate cages with trays for excreta collection at the time of sampling. No diarrhea or other clinical signs were observed. Fecal samples were collected from trays and placed into individual self-sealing bags marked with the site, age, and sex of these animals. The samples were placed in foam boxes with ice packs and transported to the laboratory. Upon arrival, they were stored at 4 °C prior to DNA extraction (not exceeding 1 week).

**Table 1:** Prevalence of *Enterocytozoon* spp. in pet hairless guinea pigs (*Cavia porcellus*) in China.

Characteristics	No. of animals	No. positive (%)	*p*-value	ITS genotypes (No.)
Sites				
Pet market (Luoyang, Henan)	35	6 (17.1)	0.0056	S7 (5), PGP (1)
Breeding facility 1 (Suzhou, Jiangsu)	72	4 (5.6)		S7 (4)
Breeding facility 2 (Xuzhou, Jiangsu)	80	7 (8.8)		S7 (7)
Breeding facility 3 (Jiangshan, Zhejiang)	65	17 (26.2)		S7 (15), PGP (2)
Breeding facility 4 (Huizhou, Guangdong)	72	12 (16.7)		S7 (11), PGP (1)
Age				
≤3 months	170	30 (17.6)	0.1094	S7 (29), PGP (1)
4–12 months	96	12 (12.5)		S7 (10), PGP (2)
>1 year	58	4 (6.9)		S7 (3), PGP (1)
Sex				
Male	141	17 (12.1)	0.3325	S7 (15), PGP (2)
Female	183	29 (15.8)		S7 (27), PGP (2)
Total	324	46 (14.2)		S7 (42), PGP (4)

### DNA extraction

Each fecal sample was mashed with a glass stick and mixed with 30 mL of distilled water. The suspension passed through a sieve (pore size of 250 μm) and was concentrated by centrifugation at 3,000 r min^−1^ for 10 min. Genomic DNA was extracted from ~200 mg processed fecal samples using an E.Z.N.A. Stool DNA Kit (Omega Biotek Inc., Norcross, GA, USA), according to the manufacturer’s instructions. The extracted DNA was kept at −20 °C until further analysis.

### PCR amplification

*Enterocytozoon bieneusi* was examined based on the ITS region by nested PCR, as previously described [1]. Two pairs of primers, EBITS3 and EBITS4, and EBITS1 and EBITS2.4 were used for the first and the second amplifications, respectively. The amplified nucleotide fragment was ~390 bp. The cycling parameters for PCRs were: 94 °C for 5 min; followed by 35 cycles of 94 °C for 30 s, 57 °C (primary PCR) or 55 °C (secondary PCR) for 30 s, and 72 °C for 40 s; and a final extension step at 72 °C for 7 min.

Selected ITS genotype PGP-positive DNA samples obtained from hairless guinea pigs in this study (*n* = 4) and regular guinea pigs in a previous study (*n* = 2) [26] were further identified by nested PCR targeting an approximately 607-bp fragment of the SSU rRNA gene. The primers were F1 (5′–CACCAGGTTGATTCTGCCTGA–3′) and R1 (5′–CCAACTGAAACCTTGTTACGACTT–3′) as external primers, and EBIEF1 (5′–GAAACTTGTCCACTCCTTACG–3′) and EBIER1 (5′–CCATGCACCACTCCTGCCATT–3′) as internal primers [3, 30]. The cycling conditions for PCRs were: 94 °C for 5 min; followed by 35 cycles of 94 °C for 45 s, 55 °C (primary PCR) or 58 °C (secondary PCR) for 45 s, and 72 °C for 1 min; followed by 72 °C for 10 min.

For the PCR amplifications mentioned above, 2×EasyTaq^®^ PCR SuperMix (TransGen Biotech, Beijing, China) was used. Positive (DNA of rat-derived genotype D) and negative (distilled water) controls were included in each PCR analysis. Secondary PCR products were examined by electrophoresis in 1.5% agarose gels and visualized after staining with GelStain (TransGen Biotech, Beijing, China).

### Sequence analysis

Positive secondary PCR products were sequenced bidirectionally by General Biol (Anhui, China). The obtained sequences were aligned with reference sequences from GenBank, using ClustalX 2.1 for SSU rRNA (http://www.clustal.org/) or MAFFT for ITS (https://www.ebi.ac.uk/Tools/msa/mafft/). The determination of the genotypes of *E. bieneusi* followed the established nomenclature system [20]. Neighbor-joining trees based on ITS and the SSU rRNA loci were generated using MEGA 7 software (http://www.megasoftware.net/). ITS sequences of *Enterocytozoon hepatopenaei* (GenBank accession no. MNPJ01000027), *Enterospora epinepheli* (OR143128), *Nucleospora salmonis* (U78176), and *Nucleospora hippocampi* (MW229243) were used as the outgroup of the ITS tree. For the tree of the SSU rRNA gene, *Enterocytozoon schreckii* (OL780325), *Enterocytozoon hepatopenaei* (FJ496356) and *Enterospora nucleophila* (KF135644) sequences were used as the outgroup. The evolutionary distances were calculated by the maximum composite likelihood model, and the reliability of branches in the trees was assessed using bootstrap analysis with 1,000 replicates.

### Statistical analysis

Chi-square analysis was performed to assess the correlation between the prevalence of *Enterocytozoon* and the age, sex, and site of pet hairless guinea pigs using SPSS, version 17.0 (Statistical Package for the Social Sciences). A difference was considered statistically significant when the *p* value was <0.05.

### Nucleotide sequence accession numbers

Representative nucleotide sequences obtained in this study were deposited in GenBank under accession numbers OQ845952–OQ845953 and OQ845783–OQ845784.

## Results and discussion

In the present study, 46 (14.2%) of 324 pet hairless guinea pigs were positive for *E. bieneusi* by PCR amplification of the ITS region. This prevalence was lower than that in pet and regular household guinea pigs in China and Peru (20.2% and 14.9%, respectively) [2, 26], but higher than in laboratory guinea pigs in China (10.9%) [27]. Many factors may affect the *Enterocytozoon* infection rates in guinea pigs, such as animal breed, age, host health condition, management and living conditions, geographical areas, and sample sizes. A significant difference of infection rates was observed among different sampling sites (*p* < 0.05), with the infection rates ranging from 5.6% to 26.2% (Table 1). The highest infection rate of *Enterocytozoon* was found in breeding facility 3, which might be due to the poor hygiene conditions. The percentage of positive animals decreased with age; the infection rate was slightly higher in females than in males (Table 1). However, no significant differences were observed between different age and sex groups (*p* > 0.05). This finding regarding the correlation between age, sex, and prevalence was in accordance with the observations in previous studies on pet chipmunks, red squirrels, hamsters, and guinea pigs in China [4, 6, 15, 26].

Two known ITS genotypes, including S7 and PGP, were identified by sequence alignment with reference sequences in the GenBank database. Genotype S7 was the predominant genotype in pet hairless guinea pigs, with a proportion of 91.3% (42/46) (Table 1). Very little is known about *E. bieneusi* in guinea pigs. A survey of regular household guinea pigs in Peru identified genotype Peru16 [2]. The other two studies were conducted in pet and laboratory regular guinea pigs in China. In pet guinea pigs, two *E. bieneusi* genotypes have been detected, including genotypes S7 and PGP, accounting for 85.7% and 14.3%, respectively [26]. In laboratory guinea pigs, only genotype S7 was found [27]. Overall, genotype S7 is the dominant genotype of *E. bieneusi* in guinea pigs, indicating that guinea pig might be an important reservoir host of genotype S7. Genotype S7 was also occasionally detected from humans, yaks, donkeys, rabbits, chipmunks, fancy rats, laboratory rats, and Asiatic brush-tailed porcupines (5 cases in chipmunk, and 1–2 each in others) [5, 6, 10–12, 22, 26].

**Figure 2:**
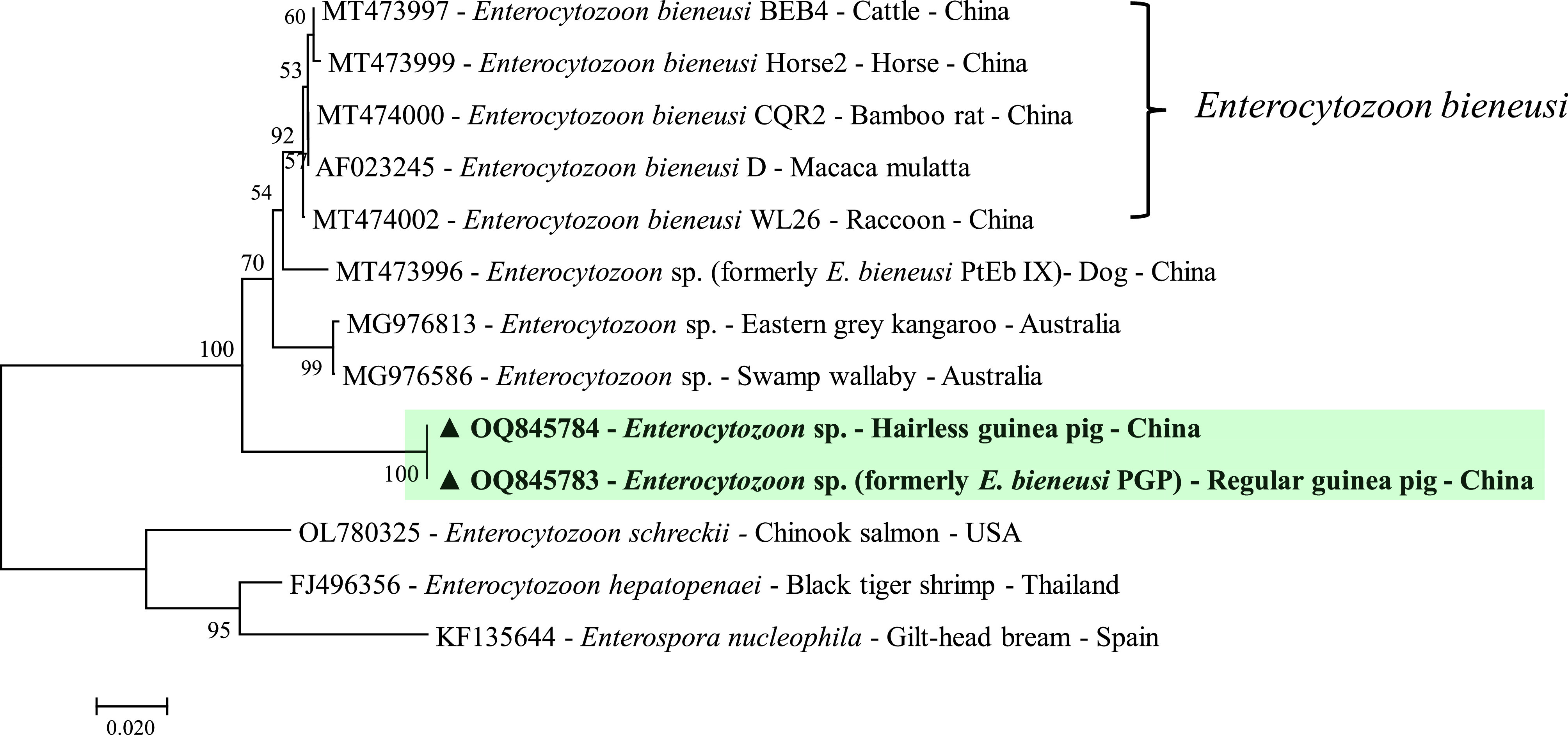
Phylogenetic tree inferred by a Neighbor-joining analysis of the partial SSU rRNA gene. Bootstrap values greater than 50% from 1,000 pseudoreplicates are shown. ▲ Representative sequences of genotype PGP.

As observed by Wang *et al*. [26], the genotype PGP had very divergent ITS sequence (248 bp) and exhibited <50% sequence similarities to the reference sequences from the known *E. bieneusi* genotype groups, canine-adapted *Enterocytozoon* sp. (also known as Group 11), and marsupial-adapted *Enterocytozoon* sp. (also known as the outliers). Given the substantial sequence divergence in ITS locus, we further used a more conserved SSU rRNA locus to study the taxonomic status of the genotype PGP. Four hairless guinea pig-derived PGP isolates and two regular guinea pig-derived isolates were selected for the identification. All the PGP isolates were PCR positive for the SSU rRNA gene, and three out of the six isolates were successfully sequenced and generated same sequences. Phylogenetic analysis of the SSU rRNA gene indicated that two representative isolates of genotype PGP formed a distinct clade far away from *E. bieneusi* (Fig. 2). They were located on a well-supported intermediate position between the marsupial-adapted *Enterocytozoon* sp. and *Enterocytozoon schreckii* (Fig. 2), with 92.0% and 85.6% similarity, respectively. This suggests that the genotype PGP represents a new species of *Enterocytozoon*.

**Figure 3:**
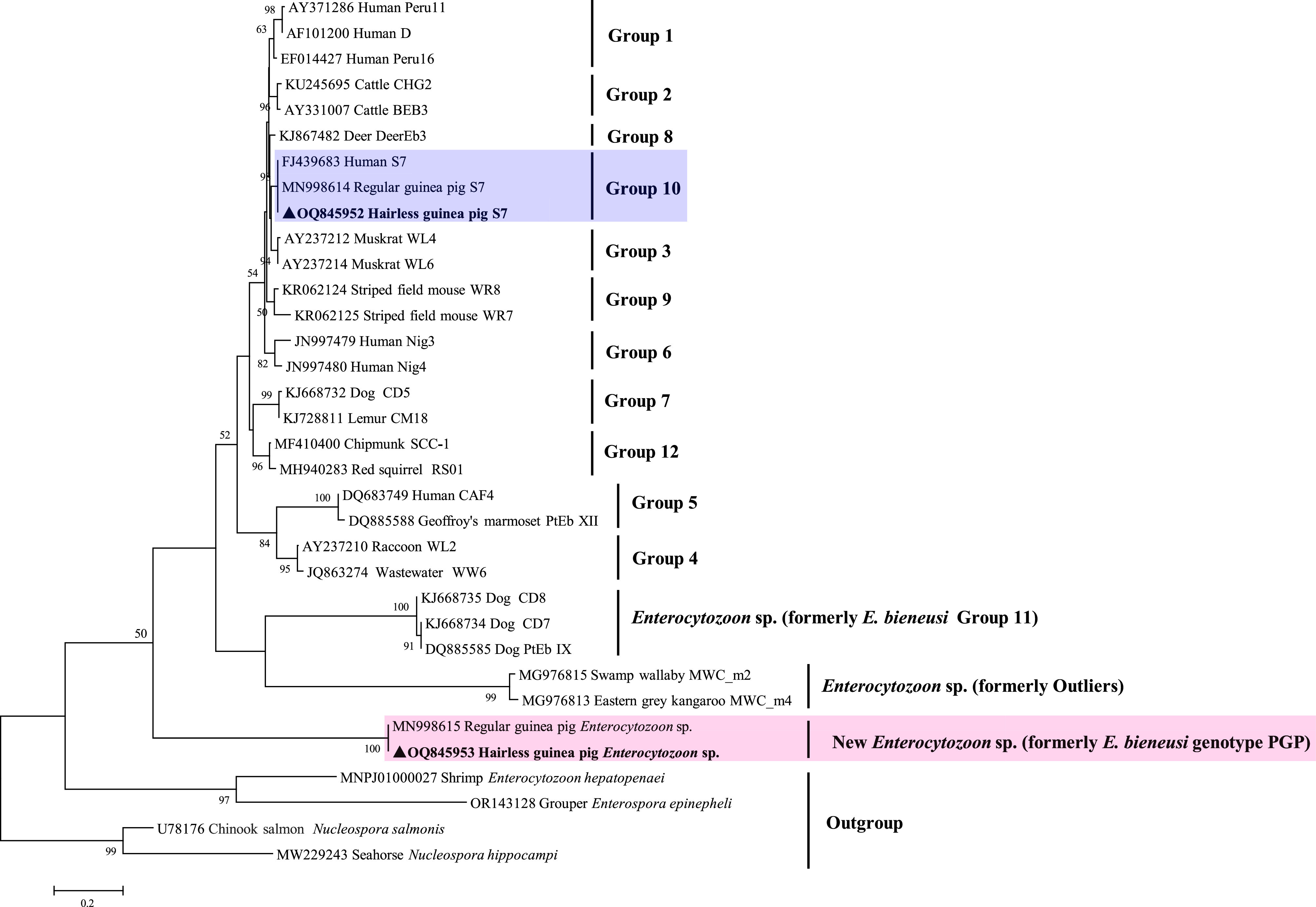
Neighbor-joining tree based on the ITS locus. Bootstrap values greater than 50% from 1,000 pseudoreplicates are shown. ▲ Genotypes identified from this study.

Generally, the sequences from the outliers and/or Group 11 were used as the outgroup or root of the *E. bieneusi* ITS tree [13, 19, 34], which may have led to the genotype PGP isolates being located on a low-supported clade among the *E. bieneusi* genotype groups and classified as a novel genotype of *E. bieneusi* in the research by Wang *et al*. [26]. In the present study, we reconstructed an ITS tree, using ITS sequences of another *Enterocytozoon* species *E. hepatopenaei*, and two related genera *Enterospora* and *Nucleospora* as the outgroup. Similar to the evolutionary relationship in the SSU rDNA tree, the PGP isolates also formed a separate clade in the ITS tree and were located between the outgroup and a large cluster consisting of canine-adapted *Enterocytozoon* sp. and marsupial-adapted *Enterocytozoon* sp., with weak bootstrap support (50%) (Fig. 3).

Previously, several ITS genotypes from marsupials and dogs were proved to have significant ITS and SSU rRNA sequence divergences from *E. bieneusi*, and thus were proposed to represent a marsupial-adapted *Enterocytozoon* sp. (formerly the outliers) and a canine-adapted *Enterocytozoon* sp. (formerly the Group 11), respectively [19, 34]. Similarly, sequence and phylogenetic analyses of the ITS and SSU rRNA loci in this study indicated that the ITS genotype PGP represents a new *Enterocytozoon* species, and it may be adapted to guinea pigs. However, when describing it as a new valid species in the future, new molecular markers are needed. In addition, spore morphology needs to be examined.

## Conclusions

This study indicates that *Enterocytozoon* infection is common in pet hairless guinea pigs. Similar to regular guinea pigs, two *Enterocytozoon* species, namely *E. bieneusi* and a new *Enterocytozoon* sp. (formerly genotype PGP) circulate in these animals in China. Zoonotic *E. bieneusi* genotype S7 was the dominant genotype, suggesting that pet hairless guinea pigs may be the potential sources of *E. bieneusi* infection in humans. For the new *Enterocytozoon* sp., more studies are needed to understand its host range and public health importance.

## References

[1] Buckholt MA, Lee JH, Tzipori S. 2002. Prevalence of *Enterocytozoon bieneusi* in swine: an 18-month survey at a slaughterhouse in Massachusetts. Applied and Environmental Microbiology, 68, 2595–2599. 11976142 10.1128/AEM.68.5.2595-2599.2002 PMC127518

[2] Cama VA, Pearson J, Cabrera L, Pacheco L, Gilman R, Meyer S, Ortega Y, Xiao L. 2007. Transmission of *Enterocytozoon bieneusi* between a child and guinea pigs. Journal of Clinical Microbiology, 45, 2708–2710. 17537930 10.1128/JCM.00725-07 PMC1951230

[3] da Silva AJ, Schwartz DA, Visvesvara GS, de Moura H, Slemenda SB, Pieniazek NJ. 1996. Sensitive PCR diagnosis of Infections by *Enterocytozoon bieneusi* (microsporidia) using primers based on the region coding for small-subunit rRNA. Journal of Clinical Microbiology, 34, 986–987. 8815125 10.1128/jcm.34.4.986-987.1996 PMC228934

[4] Deng L, Chai Y, Luo R, Yang L, Yao J, Zhong Z, Wang W, Xiang L, Fu H, Liu H, Zhou Z, Yue C, Chen W, Peng G. 2020. Occurrence and genetic characteristics of *Cryptosporidium* spp. and *Enterocytozoon bieneusi* in pet red squirrels (*Sciurus vulgaris*) in China. Scientific Reports, 10, 1026. 31974403 10.1038/s41598-020-57896-w PMC6978461

[5] Deng L, Chai Y, Xiang L, Wang W, Zhou Z, Liu H, Zhong Z, Fu H, Peng G. 2020. First identification and genotyping of *Enterocytozoon bieneusi* and *Encephalitozoon* spp. in pet rabbits in China. BMC Veterinary Research, 16, 212. 32571322 10.1186/s12917-020-02434-z PMC7310219

[6] Deng L, Li W, Zhong Z, Chai Y, Yang L, Zheng H, Wang W, Fu H, He M, Huang X, Zuo Z, Wang Y, Cao S, Liu H, Ma X, Wu K, Peng G. 2018. Molecular characterization and new genotypes of *Enterocytozoon bieneusi* in pet chipmunks (*Eutamias asiaticus*) in Sichuan province, China. BMC Microbiology, 18, 37. 29669519 10.1186/s12866-018-1175-y PMC5907217

[7] Gui BZ, Zou Y, Chen YW, Li F, Jin YC, Liu MT, Yi JN, Zheng WB, Liu GH. 2020. Novel genotypes and multilocus genotypes of *Enterocytozoon bieneusi* in two wild rat species in China: potential for zoonotic transmission. Parasitology Research, 119, 283–290. 31811423 10.1007/s00436-019-06491-8

[8] Han B, Pan G, Weiss LM. 2021. Microsporidiosis in Humans. Clinical Microbiology Reviews, 34, e0001020. 34190570 10.1128/CMR.00010-20 PMC8404701

[9] Hu B, Wang J, Zhang S, Wang B, Xing Y, Han S, He H. 2022. Novel genotypes of *Cryptosporidium* and *Enterocytozoon bieneusi* detected in plateau zokors (*Myospalax baileyi*) from the Tibetan Plateau. International Journal for Parasitology: Parasites and Wildlife, 19, 263–268. 36388721 10.1016/j.ijppaw.2022.11.002 PMC9661441

[10] Li F, Wang R, Guo Y, Li N, Feng Y, Xiao L. 2020. Zoonotic potential of *Enterocytozoon bieneusi* and *Giardia duodenalis* in horses and donkeys in northern China. Parasitology Research, 119, 1101–1108. 32006227 10.1007/s00436-020-06612-8

[11] Li J, Jiang Y, Wang W, Chao L, Jia Y, Yuan Y, Wang J, Qiu J, Qi M. 2020. Molecular identification and genotyping of *Enterocytozoon bieneusi* in experimental rats in China. Experimental Parasitology, 210, 107850. 32027893 10.1016/j.exppara.2020.107850

[12] Li J, Qi M, Chang Y, Wang R, Li T, Dong H, Zhang L. 2015. Molecular Characterization of *Cryptosporidium* spp., *Giardia duodenalis*, and *Enterocytozoon bieneusi* in Captive Wildlife at Zhengzhou Zoo, China. Journal of Eukaryotic Microbiology, 62, 833–839. 26384582 10.1111/jeu.12269

[13] Li W, Feng Y, Santin M. 2019. Host specificity of *Enterocytozoon bieneusi* and public health implications. Trends in Parasitology, 35, 436–451. 31076351 10.1016/j.pt.2019.04.004

[14] Li W, Xiao L. 2021. Ecological and public health significance of *Enterocytozoon bieneusi*. One Health, 12, 100209. 33426263 10.1016/j.onehlt.2020.100209 PMC7779778

[15] Lv C, Wang J, Li C, Zhang M, Qian W. 2022. First detection and genotyping of *Enterocytozoon bieneusi* in pet golden hamsters (*Mesocricetus auratus*) and Siberian hamsters (*Phodopus sungorus*) in China. Parasite, 29, 15. 35315766 10.1051/parasite/2022018 PMC8939298

[16] Masuda A, Wada M, Saho H, Tokunaga K, Kikuchi Y, Yamasaki F, Matsumoto J. 2021. Prevalence and molecular characterization of the zoonotic enteric protozoans *Cryptosporidium* spp., *Enterocytozoon bieneusi*, and *Blastocystis* from Pallas’s Squirrels (*Callosciurus erythraeus*) in Kanagawa Prefecture, Japan. Microbiology Spectrum, 9, e0099021. 34730381 10.1128/Spectrum.00990-21 PMC8567245

[17] Meerburg BG, Singleton GR, Kijlstra A. 2009. Rodent-borne diseases and their risks for public health. Critical Reviews in Microbiology, 35, 221–270. 19548807 10.1080/10408410902989837

[18] Ni HB, Sun YZ, Qin SY, Wang YC, Zhao Q, Sun ZY, Zhang M, Yang D, Feng ZH, Guan ZH, Qiu HY, Wang HX, Xue NY, Sun HT. 2021. Molecular detection of *Cryptosporidium* spp. and *Enterocytozoon bieneusi* infection in wild rodents from six provinces in China. Frontiers in Cellular and Infection Microbiology, 11, 783508. 34900760 10.3389/fcimb.2021.783508 PMC8656357

[19] Ou Y, Jiang W, Roellig DM, Wan Z, Li N, Guo Y, Feng Y, Xiao L. 2021. Characterizations of *Enterocytozoon bieneusi* at new genetic loci reveal a lack of strict host specificity among common genotypes and the existence of a canine-adapted *Enterocytozoon* species. International Journal for Parasitology, 51, 215–223. 33275946 10.1016/j.ijpara.2020.09.008

[20] Santin M, Fayer R. 2009. *Enterocytozoon bieneusi* genotype nomenclature based on the internal transcribed spacer sequence: a consensus. Journal of Eukaryotic Microbiology, 56, 34–38. 19335772 10.1111/j.1550-7408.2008.00380.x

[21] Tavalla M, Kazemi F, Mardani-Kateki M, Abdizadeh R. 2018. Molecular diagnosis of *Enterocytozoon bieneusi* and *Encephalitozoon* spp. in wild rats of southwest of Iran. Jundishapur Journal of Microbiology, 11, e55961.

[22] ten Hove RJ, Van Lieshout L, Beadsworth MB, Perez MA, Spee K, Claas EC, Verweij JJ. 2009. Characterization of genotypes of *Enterocytozoon bieneusi* in immunosuppressed and immunocompetent patient groups. Journal of Eukaryotic Microbiology, 56, 388–393. 19602086 10.1111/j.1550-7408.2009.00393.x

[23] Venturo R. 2021. Hair loss in guinea pigs. Canadian Veterinary Journal, 62, 77–80. PMC7739396 33390607

[24] Vioque F, Dashti A, Santin M, Ruiz-Fons F, Koster PC, Hernandez-Castro C, Garcia JT, Bailo B, Ortega S, Olea PP, Arce F, Chicharro C, Nieto J, Gonzalez F, Vinuela J, Carmena D, Gonzalez-Barrio D. 2022. Wild micromammal host spectrum of zoonotic eukaryotic parasites in Spain. Occurrence and genetic characterisation. Transboundary and Emerging Diseases, 69, e2926–e2942. 35752461 10.1111/tbed.14643

[25] Wang H, Liu Q, Jiang X, Zhang Y, Zhao A, Cui Z, Li D, Qi M, Zhang L. 2019. Dominance of zoonotic genotype D of *Enterocytozoon bieneusi* in bamboo rats (*Rhizomys sinensis*). Infection, Genetics and Evolution, 73, 113–118. 10.1016/j.meegid.2019.04.025 31029793

[26] Wang J, Lv C, Zhao D, Zhu R, Li C, Qian W. 2020. First detection and genotyping of *Enterocytozoon bieneusi* in pet fancy rats (*Rattus norvegicus*) and guinea pigs (*Cavia porcellus*) in China. Parasite, 27, 21. 32249756 10.1051/parasite/2020019 PMC7133117

[27] Wang N, Wang K, Liu Y, Zhang X, Zhao J, Zhang S, Zhang L. 2022. Molecular characterization of *Cryptosporidium* spp., *Enterocytozoon bieneusi* and *Giardia duodenalis* in laboratory rodents in China. Parasite, 29, 46. 36219067 10.1051/parasite/2022046 PMC9552759

[28] Wang SS, Wang RJ, Fan XC, Liu TL, Zhang LX, Zhao GH. 2018. Prevalence and genotypes of *Enterocytozoon bieneusi* in China. Acta Tropica, 183, 142–152. 29660311 10.1016/j.actatropica.2018.04.017

[29] Wu D, de Linde Henriksen M, Grant K, Lyakhova T, Sharp JL, Daniels JB. 2020. Ocular findings and selected ophthalmic diagnostic tests in a group of young commercially available Guinea and Skinny pigs (*Cavia porcellus*). Veterinary ophthalmology, 23, 234–244. 31562703 10.1111/vop.12709

[30] Xiao L, Li L, Moura H, Sulaiman IM, Lal AA, Gatti S, Scaglia M, Didier ES, Visvesvara GS. 2001. Genotyping *Encephalitozoon* parasites using multilocus analyses of genes with repetitive sequences. Journal of Eukaryotic Microbiology, 48, S1, 63S–65S. 10.1111/j.1550-7408.2001.tb00454.x 11906081

[31] Xu J, Wang X, Jing H, Cao S, Zhang X, Jiang Y, Yin J, Cao J, Shen Y. 2020. Identification and genotyping of *Enterocytozoon bieneusi* in wild Himalayan marmots (*Marmota himalayana*) and Alashan ground squirrels (*Spermophilus alashanicus*) in the Qinghai-Tibetan Plateau area (QTPA) of Gansu Province, China. Parasites & Vectors, 13, 367. 32698833 10.1186/s13071-020-04233-9 PMC7376879

[32] Yu F, Cao Y, Wang H, Liu Q, Zhao A, Qi M, Zhang L. 2020. Host-adaptation of the rare *Enterocytozoon bieneusi* genotype CHN4 in *Myocastor coypus* (Rodentia: Echimyidae) in China. Parasites & Vectors, 13, 578. 33198788 10.1186/s13071-020-04436-0 PMC7667729

[33] Yu F, Qi M, Zhao Z, Lv C, Wang Y, Wang R, Zhang L. 2019. The potential role of synanthropic rodents and flies in the transmission of *Enterocytozoon bieneusi* on a dairy cattle farm in China. Journal of Eukaryotic Microbiology, 66, 435–441. 30191674 10.1111/jeu.12687

[34] Zhang Y, Koehler AV, Wang T, Haydon SR, Gasser RB. 2018. New operational taxonomic units of *Enterocytozoon* in three marsupial species. Parasites & Vectors, 11, 371. 29954462 10.1186/s13071-018-2954-x PMC6022301

[35] Zhao W, Wang J, Ren G, Yang Z, Yang F, Zhang W, Xu Y, Liu A, Ling H. 2018. Molecular characterizations of *Cryptosporidium* spp. and *Enterocytozoon bieneusi* in brown rats (*Rattus norvegicus*) from Heilongjiang Province, China. Parasites & Vectors, 11, 313. 29793513 10.1186/s13071-018-2892-7 PMC5968579

[36] Zhao W, Wang T, Ren G, Li J, Tan F, Li W, Zhu C, Lu G, Huang H. 2023. Molecular detection of *Enterocytozoon bieneusi* in farmed Asiatic brush-tailed porcupines (*Atherurus macrourus*) and bamboo rats (*Rhizomys pruinosus*) from Hainan Province, China: common occurrence, wide genetic variation and high zoonotic potential. Acta Tropica, 242, 106915. 36997011 10.1016/j.actatropica.2023.106915

[37] Zhao W, Zhou H, Yang L, Ma T, Zhou J, Liu H, Lu G, Huang H. 2020. Prevalence, genetic diversity and implications for public health of *Enterocytozoon bieneusi* in various rodents from Hainan Province, China. Parasites & Vectors, 13, 438. 32878633 10.1186/s13071-020-04314-9 PMC7466830

